# The Dreyer Tuberculosis Vaccine

**Published:** 1923-09

**Authors:** 


### THE DREYER TUBERCULOSIS VACCINE.

PROFESSOR DREYER'S diaplyte vaccine is not at present quite so popular as it was on its introduction a few months ago. This does not necessarily mean that it is faulty and that its faults are now being found out. But it would be well at the present stage to point out that the opinions of those physicians who have already had practical experience of this vaccine are not unanimously in its favour. True, it does not seem to be as dangerous as the old tuberculin which, in large doses, has been held responsible for many disasters. On the other hand, several cases of pulmonary tuberculosis given Dreyer's vaccine have shown little or no improvement, and one of the questions now being asked is: Have the patients given this treatment been selected with adequate discrimination? It is obviously unfair to the reputation of the vaccine to give it exclusively to moribund patients in the third and last stage of the disease. If, however, only first-stage cases had been selected for a trial of the new vaccine, and if recovery had followed, the inevitable judgment would have been: "Patients in the first stage usually recover without any treatment." Again, there is the question of dosage.

An Experimental Stage.

Little is at present known of the optimum dose of the vaccine, and the amount given to patients is, weight for weight, many times less than that given to the guinea-pigs which have responded satisfactorily to the vaccine. It is conceivable that the dosage at present in use will in a year or two be regarded as timid and altogether inadequate. But it is better to proceed from small to large doses in the experimental stage than to begin by large doses which may do harm not only to the patient, but to the good name of the vaccine. Already certain modifications have been adopted in the manufacture of the vaccine, and it is being given searching and extensive trial both in man and in experimental animals. Professor Dreyer has gone to Denmark with a supply which will be tested by some of his own countrymen at the Finsen Institute in Copenhagen. With searching tests at home and abroad, it is to be hoped that in a year or two it will be possible to form a definite opinion as to the merits of this treatment. At present we do not know whether it is to be depended upon or not, and we have certainly much to learn with regard to dosage and the spacing of the intervals between each injection. It is essential that the many patients now clamouring for the treatment should realise its unproven character.

